# Challenges for group leaders working with families dealing with early psychosis: a qualitative study

**DOI:** 10.1186/s12888-015-0540-8

**Published:** 2015-07-02

**Authors:** Liv Nilsen, Irene Norheim, Jan C. Frich, Svein Friis, Jan Ivar Røssberg

**Affiliations:** 1 Centre of Competence for Early Intervention in Psychosis, Division of Mental Health and Addiction, Oslo University Hospital, Oslo, Norway; 2 Division of Mental Health and Addiction, Vestre Viken Hospital Trust, Drammen, Norway; 3 Institute of Health and Society, University of Oslo, Oslo, Norway; 4 Division of Mental Health and Addiction, Oslo University Hospital, Oslo, Norway; 5 Institute of Clinical Medicine, University of Oslo, Oslo, Norway; 6 KG Jebsen Centre for Psychosis Research, Division of Mental Health and Addiction, Oslo University Hospital, Oslo, Norway

**Keywords:** Early intervention, Family work, Psychosis, Qualitative research

## Abstract

**Background:**

Family work is one of the best researched psychosocial interventions for patients with chronic psychosis. However, family work is less studied for patients with a first episode psychosis and the studies have revealed contradicting results. To our knowledge, no previous studies have examined qualitatively group leaders’ experiences with family work. In the present study we wanted to explore challenges faced by mental health professionals working as group leaders for family interventions with first episode psychosis patients.

**Method:**

A qualitative exploratory study was carried out based on digitally recorded in-depth interviews and a focus group interview with nine experienced mental health professionals. The interviews were transcribed in a slightly modified verbatim mode and analysed by systematic text condensation.

**Results:**

Challenges faced by group leaders was classified into six categories: (1) Motivating patients to participate, encouraging potential participants was demanding and time-consuming; (2) Selecting participants by identifying those who can form a functional group and benefit from the intervention; (3) Choosing group format to determine whether a single or multi-family group is best for the participants; (4) Preserving patient independence, while also encouraging them to participate in the intervention; (5) Adherence to the protocol, while customizing adjustments as needed; (6) Fostering good problem-solving by creating a fertile learning environment and choosing the most appropriate problem to solve.

**Conclusions:**

Group leaders face challenges related to recruitment and selection of participants for family work, as well as in conducting sessions. Awareness of these challenges could help health professionals more specifically to tailor the intervention to the specific needs of patients and their families.

## Background

Onset of psychotic disorders typically occurs in late adolescence or early adulthood [1]. Although the course of psychosis may vary substantially among patients, many patients have poor long-term outcomes [2] resulting in personal suffering and costs to society. Over the past two decades the major focus has been on early intervention, with the primary aim of initiating treatment (e.g., antipsychotics, family work and individual psychotherapy) as early as possible to reduce the severity of symptoms and increase psychosocial functioning. The effect of antipsychotic medication in reducing psychotic symptoms and the risk of relapse are well documented [3]. However, a large majority of patients require additional psychosocial intervention. The efficacy of family intervention for individuals experiencing psychotic symptoms for several years is well documented [4]. However, to our knowledge there are only five quantitative studies on first episode psychosis (FEP) and family interventions. The results are contradictory; two studies showed positive effect [5, 6], two studies showed negative effect [7, 8] and one study showed no effect [9].

Regarding qualitative studies, in a descriptive review of studies between 1996 and 2008, Boydell and colleagues found no studies about family work from the mental health perspective [10]. To our knowledge no other studies have been published on this topic since 2008. Our group recently [11, 12] published two studies examining the perspective of patients and family members on participation in family intervention. The first study [11] examined the reported experiences of patients and family members with family intervention, which demonstrated that a good relationship with group leaders was important to avoid participant attrition. Furthermore, the study revealed that meeting other people in the same situation reduced feelings of shame and increased hope for the future. Narratives from real life were considered to be more important sources of knowledge about psychosis than lectures and workshops, but many patients experienced considerable anxiety and tension during meetings. The group format could be difficult for patients immediately after a psychotic episode, and for those still struggling with distressing psychotic symptoms. The second study [12] examined how patients and family members perceived the benefits of participating in family intervention. Family intervention benefits included gaining insight and acceptance of the illness. Moreover, it was important to recognize warning signs and take them into account, as well as to learn new ways to communicate. The patients also felt that they gained more independence and were able to take responsibility for their own lives.

Patients with a first episode psychosis and their families are best qualified to describe the family intervention experience. However, health professionals can probably offer important additional information. They may be experienced in aspects of interventions that are useful for assessing patient potential and aiding progress towards recovery. Challenges, experienced by health professionals conducting family work, could be to decide what kind of patients they should invite to participate, what kind of relatives, when in the illness process they should invite the participants to join family work, should patients and family members participate together, how strict must the group leaders follow the treatment manual and how could they best evaluate how much stress and anxiety the patients are able to tolerate? These difficulties and challenges, in conducting family work are, to our knowledge, never explored in earlier studies. As health professionals and researchers experienced in psychiatry and family work, we were interested in learning more about these challenges in order to improve the intervention for patients with a FEP and their families.

### Aim of the study

This study aims to explore the challenges faced by mental health professionals as group leaders in family interventions for first episode psychosis patients.

## Methods

This qualitative exploratory study, conducted in a natural setting, is based on data from in-depth single interviews and a focus group interview. As the goal was to explore challenges in conducting family work, a qualitative approach was chosen. The study was carried out at the Centre of Competence for Early Intervention in Psychosis (TIPS), and is part of the Thematic Research area Psychosis (TOP) study at the University of Oslo and Oslo University Hospital.

### Participants

Mental health professionals trained in psychoeducational family work, based on the manual developed by McFarlane and colleagues [13, 14], were recruited from hospitals in southeast Norway. Fifteen health professionals were invited to a focus group interview. Six did not respond to the invitation and four were not able to participate. To achieve a more heterogeneous material we invited the four group leaders, who were not able to join the focus group interview, to individual in depth interviews. The sampling strategy aimed at achieving diversity of health professionals; the sample included psychiatric nurses, occupational therapists, psychologists and psychiatrists with group leader experience. A total of nine mental health professionals agreed to participate and provided informed consent. All but one had conducted sessions with one or two multi-family groups, while four had experience with 1 to 14 single-family groups. The length of intervention was one year for single- family groups and two years for multi- family groups. All participants were women with five to 15 years of experience in family work. The number of interviewees was small, but hopefully the participants’ long and varied clinical experience compensates for that. During the interview process it became evident that the group leader experienced challenges in conducting family work. It was easily discussed, and the material revealed answers about their perceived challenges, especially during the recruitment phase. Their concerns were mainly about how they best could take care of the young patients in a difficult situation. In spite of the low numbers of participants, in the present study, we secured saturation by performing the analysis concurrently with the data collection and by continuously evaluating the interviews and the transcripts.

### Data collection

An interview guide was developed in cooperation with patients, family members and health professionals familiar with the intervention. The guide was based on the manual and efforts were made to ensure coverage of all elements of the intervention (Table 1).

**Table 1:** Interview guide

● How would you describe your experience as a group leader?
● The treatment is divided into phases, could you describe your experiences with the different phases. Obstacles, challenges and positive experiences.
○ The joining in period
○ The survival skills work shop
○ The meetings
● What are the most challenging subjects in conducting family work?
● How do you differentiate between those who should be offered a single- or a multi-family intervention?
● What benefits do you think the participants experienced by participating in the intervention?
● Is there something within the intervention that makes it easy/difficult to participate?
● What changes should be made to make the intervention more beneficial for patients experiencing a first episode psychosis?
● How would you describe the patients who drop out?

The first author conducted the interviews, which lasted between 30 and 110 min, between June and November 2013. The second author (IN) participated as an assistant moderator in the focus group interview. The interviews were digitally recorded, and transcribed in a slightly modified verbatim mode [15] by the first author. Both LN and IN are psychoeducational multi-family group (PEMFG) leaders with experience conducting and supervising the intervention with patients suffering from various psychotic disorders. This may have influenced the results. However, all authors made a deliberate effort to bracket preconceptions by having an interdisciplinary dialogue throughout the research process.

### The intervention

Psycho educational family work is a method for working with families who have a member suffering from mental illness. The goals are to improve outcome and quality of life as well as to reduce family stress and strain and has a multi-family or a single-family format [13, 16]. The method comprises three stages: the joining in period, survival skills workshop and the meetings. The meetings are usually biweekly and last for 90 min in a multi family approach and for 45 min in a single family intervention. The intervention is originally designed for patients suffering from long lasting mental disorders, but is also an intervention recommended for patients with a FEP [8, 17].

### Ethical aspects

The study was approved by the Regional Committee for Medical and Health Research Ethics for southeast Norway (REC South East) (2011/566).

### Analysis

Data were analysed according to the principles of systematic text condensation (STC) [18]. Analysis was conducted in four steps, and steps two and three were analysed using NVivo 10. First we read through the interviews to achieve an overall impression, and to look for preliminary themes related to the challenges faced by mental health professionals working with patients and family members in psychoeducational family intervention. Second, we broke down the text into manageable meaning units and connected related meaning units into code groups. Third, we condensed the meaning under each code group. Fourth, we developed an analytic text about the six categories we found relevant for this study. The first and the last author read through all the interviews separately several times and identified meaning units. All authors were involved in the analysis, determining the categories and their content. Agreement was reached through group discussion.

## Results

Health professionals reported six challenges (Table 2): (1) Motivating patients to participate in the intervention, because encouraging potential participants was both demanding and time-consuming; (2) Selecting participants by identifying those who can form a functional group and benefit from the intervention; (3) Choosing group format to determine whether a single or multi-family group is best for the patients and their families; (4) Preserving patient independence, while also encouraging them to participate in the intervention; (5) Adherence to protocol, while customising adjustments as needed; (6) Fostering good problem-solving by creating a fertile learning environment and choosing the most appropriate problem for patients to solve.

**Table 2:** Important challenges emphasised by mental health professionals conducting psychoeducational family interventions in early psychosis

Theme	Quotes from mental health professionals
Motivating patients to participate	“The recruitment period starts very early and it is necessary to take small steps to avoid frightening the patients away.”
	“I had to put my heart into the work; I had to say that I really believe this intervention is something worth trying…I know it has been useful for others in the same situation.”
	“Patients get a lot of offers and you have to promote the intervention.”
	“For some patients it took a year before they were ready to accept the invitation.”
	“It was much more difficult to recruit patients into a group than I would have thought.”
Selecting participants	“In the future I would have been much more responsive to patients who do not want to participate.”
	“This type of family work is an important part of treatment for psychosis, and it feels like a loss when someone drops out. But it isn’t right for everyone.”
	“Looking back, I think we exposed some patients to too much pressure during the recruitment phase, the family members were motivated, but the situation caused substantial anxiety for the patient.”
	“I think he [the patient] became traumatized and it hurts to think about it…In the future I will listen to my clinical experience.”
	“I don’t think it is right to bring people from different life situations and with different types of illness, symptoms and needs into the same group.”
Choosing group format	“Those who are able to identify themselves as having an illness, and at the same time are able to distance themselves from feelings of loss and sorrow, gained more from participating in a group …the ones caught up in their emotions became anxious.”
	“In a single-family group, family secrets could have been revealed. This would give the family members opportunities to talk about issues they never have discussed before.”
	“It was difficult to handle the group, especially when some family members talked too much or ignored the structure.”
Preserving patient independence	“Patients often feel embarrassed participating in an intervention together with their family members; they hardly want their family to participate in an ordinary treatment session.”
	“Patients prefer to keep up with their usual activities and to maintain their normal life.”
	“If the patients’ capacity is to be social twice a week, they prefer to be with friends rather than in a group.”
Adherence to protocol	“…if you are unfamiliar with the method, the manual could be something to hold on to.”
	“You have to be flexible and make use of your clinical experience, not strictly follow the manual.”
	“You have to be yourself and communicate in a language and in words you feel comfortable with.”
	“The ability to look above and beyond the manual makes you a good group leader.”
Fostering good problem-solving	“Being able to explore together in the group and realising that they [the patients] were able to handle the problem”
	“I think a more optimistic view … and talking about hope, achievements and resources, would have suited the participant better than talking about problems“
	“The opportunity to ask about what is going well is the brilliant part of this intervention, which improved conversations. Otherwise it could have been difficult to handle”

### Motivating patients to participate

In the recruitment phase, health professionals faced challenges in motivating patients. Despite the heavy caseload of potential participants, motivating patients to participate in multi-family group intervention was experienced as time-consuming and intense. While most family members were motivated and willing to participate immediately, patients were anxious about the intervention, which frequently caused considerable delays before consent was given. Understanding these feelings was important in the recruitment process. Experience as a group leader could be a strength for motivating potential participants, since they could refer to their own confidence in the intervention, citing experiences from previous participants. The interviewees argued that group leaders should be involved in the recruitment phase from the start, in order to establish good alliance with participants. In their experience, participants who showed little or no interest during the bonding period were more likely to drop out of the intervention.

### Selecting participants

Health professionals emphasized that multi-family group interventions were not appropriate for all eligible participants. They found that patients often were reluctant to participate in such a long lasting intervention. They experienced that patients were eager to return to their ordinary lives and were not interested in further treatment. The health professionals felt that high-pressure persuasive techniques during recruitment could traumatize vulnerable patients. This became especially evident for patients with a short period of illness or with rapid remission. They were not interested in the intervention even though the families were eager to participate. For future purposes, health professionals concluded that they needed to be more responsive to the unwillingness of some patients to participate, but found it challenging because they wanted to provide patients with a treatment they found beneficial for most patients. They concluded that there are many paths to recovery and that this particular intervention might not be suitable for all FEP patients and their families.

### Choosing group format

Deciding whether to include participants in single or multi-family intervention could be challenging. The ability of participants to manage troublesome and difficult feelings was important. The health professionals realized that not all participants would tolerate being with others who were perhaps more ill, or with those in a more stable recovery phase. In such situations, single-family intervention might be the best choice. Health professionals found that vulnerable patients who became anxious tended to drop out of the intervention. Those who accepted their mental disorder while managing to control their feelings of loss and sorrow gained more from participation. Some family members suffered from symptoms themselves or had such serious and difficult problems that participation in multi-family group intervention was unsuitable. Health professionals familiar with both single and multi-family group intervention felt that families were able to discuss more serious issues in a single-family group. The recruitment process usually helped to differentiate between participants who would benefit from single-family group intervention and those who would benefit from multi-family group intervention.

### Preserving patient independence

Young people experiencing their first episode of psychosis are often at an age where fitting in with peers is important. They are often in a separation process and prefer to spend their time with friends rather than participate in family intervention. At the same time the family is struggling to let go of their offspring at a time when they perceive that something is wrong. Health professionals experienced this tension between the desire to be a “healthy normal” person and the difficulties caused by the illness to be a challenge. On the one hand they know that intervention could benefit both patient and family, while on the other hand they realize that participants must accept the need for treatment so they will participate in the intervention.

### Adherence to protocol

Health professionals described the manual as a useful guide that enabled them to work systematically. One challenge was how to remain flexible within the set guidelines. They realised that to be a good clinicians they had to customise the intervention to the situation; otherwise the solution would not be a good fit. They were also concerned about adherence to the manual. They found it difficult to know when they were in line with the model and when they crossed that line.

### Fostering good problem-solving

At each treatment session, health professionals choose a problem from one of the patients to solve. Participants usually suggested a number of answers for each specific problem. The health professionals found it difficult to narrow down the number of suggestions they received. However, when they broke the problems down into manageable pieces, participants learned new ways to solve their personal problems. The health professionals also noted the importance of the problem-solving method in reducing tension and anxiety within the group. Patients often preferred to talk about what they had already accomplished, rather than about their remaining problems. Similarly, family members preferred to talk about issues that were going well. The ability to do so was considered to be a strength of the intervention and often succeeded in easing tense situations. Health professionals found it difficult to decide whether they should focus on patient problems or patient accomplishments during the session; clinical experience was considered to be of major importance in this regard.

## Discussion

We found that the challenges faced by group leaders could be classified into six categories: Motivating patients to participate, selecting participants, choosing group format, preserving patient independence, adherence to protocol and fostering good problem-solving.

### Motivating patients to participate

Our results suggest that participant motivation and the experience and skills of the health professional were essential during the recruitment phase. To communicate information in an easy and understandable way has been reported as important in a study concerning behavioural family therapy [19]. This is in line with the findings in the present study that included patients with a FEP. In a study investigating factors for engagement in the initial stages of treatment, Stewart [20] found several essential factors such as the ability of health professionals to provide education about the illness, to provide guidance through treatment, the ability to identify and support patients’ personal strengths as well as to present an optimistic view of the future with a focus on the individual rather than on the illness. Our findings are consistent with these results, suggesting that the quality of the relationship during the recruitment process is important for successful engagement into treatment for young persons with FEP. In our study, group leaders found that family members usually were motivated at an earlier stage than patients. Stewart found that the patient decision to remain in treatment was driven by accepting and engaging in relationships with health professionals [20]. Our findings underscore that relational competence is crucial for mental health professionals who embark on training programs to become group leaders for psychoeducational group work.

### Selecting participants

Group leaders found that selecting participants could be a challenge, and we noted tension between the use of persuasion by group leaders and reluctance from the patient to participate. Patients experiencing FEP may not be at a stage in their illness where they understand the need for treatment. A two-year intervention that involves sharing experiences with others may cause ambivalence and anxiety. Although most families were eager to participate, some were more reluctant. Interestingly, this finding is in line with well-known barriers to recruiting patients with FEP into research projects. Furimsky and colleagues [21] noted that patients in an early stage of illness need to develop insight and acceptance of their diagnosis before consenting to participate in research projects. Moreover, some family members work full-time and may be unable to take time off to participate. Gonzalez and Steinglass [22] showed that the intervention should be timed to coincide with the needs of participants, the demands of the situation and the different phases of the illness. They referred to conditions such as diabetes and cystic fibrosis as diseases that require about two years for patients to accept, and they state that it is likely that FEP patients and their families require the same length of time. Our study adds to previous knowledge by underscoring the conclusion that patients with a psychotic disorder need time to reach a state of acceptance.

### Choosing group format

Group leaders reported challenges in choosing patients and family members that could work together in an optimal and meaningful way. Some of the patients were too vulnerable to participate in a multi-family group, and some family members suffered from symptoms that were too serious for them to participate. These families were more likely to benefit from a single-family intervention approach. The manual describing the intervention claims that single-family interventions have been found to be more effective for patients who respond positively to medication and whose families are emotionally resilient and have already adopted good coping skills. Multi-family groups are effective in patients and families with more severe disabilities [13]. This is consistent with the findings of our group [11] in a previous study: the decision on whether to participate in single or multi-family intervention should be individualised during the relationship-building phase, depending on social skills and intensity of distressing symptoms. This might describe an important difference between working with FEP and working with those suffering from chronic psychosis. This knowledge is important in order to offer the right treatment to the right person at the right time.

### Preserving patient independence

We found that group leaders experience tension between preserving patient independence and encouraging patients to participate in family intervention. Patients often experience their first episode of psychosis at a time when personality development and identity issues are likely to manifest and when the separation phase is underway. Nevertheless, they still depend on their families for housing, money and transportation. Moreover, families are an important part of the social network for young people who develop a psychosis [21]. In a study by Windell and colleagues [23], patients with FEP described that “hope-inspiring” health professionals could be enormously influential by reducing stigma and increasing acceptance of being ill and the need for treatment. This is in line with the findings of our study, where health professionals had to achieve balance between their knowledge about how helpful such an intervention might be and the struggles of participants to maintain their everyday life. Understanding this dilemma and how to manage it are important for optimal handling of these issues during the recruitment phase.

### Adherence to protocol

Our results indicate that health professionals used the manual as a guide that enabled them to work systematically, but they had concerns about how to use the manual in a flexible way so as to accommodate the individual circumstances of participants. This is in line with previous studies that have included patients with chronic mental illnesses. Mental health professionals and families valued a clear structure, but they also wanted flexibility in conducting the intervention [19, 24]. The family intervention is evidence-based [25, 26], according to studies in research settings [27–29]. In those settings the treatment manual must be strictly followed. Our study suggests that group leaders should balance rigour and flexibility in their clinical approach, which is in line with the above mentioned studies [19, 24], and Nock *et al.*, who described the flexible use of evidence-based treatment [30]. Knowledge and clinical skills in how to individualise treatment within the guidelines of the manual are important in order to provide all participants with the best possible family treatment.

### Fostering good problem-solving

Problem-solving was linked to challenges faced by group leaders in choosing the most appropriate problems for patients to solve while creating a good learning environment, characterised by an acceptable anxiety level, that stimulates improvement by solving problems in a constructive and meaningful way. Norman and colleagues [31] found that participants emphasised the value of health professionals who provide helpful information and remain hopeful, while customising their therapy to meet the particular needs of the situation. Helpful information should be provided within the context of enhancing and expanding the patients’ level of choice. Relationships with others suffering from the same illness provided participants with useful information about coping strategies allowing them to gain more control over their own situations. This is in line with health professionals in our study who emphasise the importance of a positive and optimistic view as essential for the recovery process. Pihet and colleagues [32] found that when participants experienced success, treatment motivation also increased. Our study indicates that a success factor for recovery might be the ability to handle problems in a meaningful way, and that the creation of an optimal treatment environment in the group is likely to be of major importance for patient improvement.

### Limitations and strengths

The scope of the present study was to explore challenges related to family interventions from the perspective of group leaders. Although the study has a small sample size, we believe that the long and varied experience of participants compensates for this. Although several men were invited to participate in the study, our sample consists only of women, and our sample may therefore be associated with gender bias. Still, the challenges our participants report are not specifically related to the gender of the group leaders, and we believe that our findings reflect the experiences of both male and female group leaders. The first and the second author are experienced group leaders who have been conducting family work and have supervised group leaders for several years. They both share a theoretical approach that is consistent with McFarlane’s manual. While this may have influenced the results, the research group made a deliberate effort to bracket preconceptions in all phases of the study. Still, it is possible that researchers working with a different theoretical framework might have identified and classified themes differently than what was done in the present study. The results may not be transferable to all participants with FEP; therefore knowledge about the results might be of importance to help group leaders to avoid some of the pitfalls in facilitating the intervention.

## Conclusion

Group leaders face challenges related to recruitment and selection of participants for family work, as well as those related to conducting sessions. Awareness of these challenges and strategies to manage them could help professionals to successfully tailor interventions to patients and their families.
